# Colony-stimulating factor 1 receptor inhibition prevents disruption of the blood-retina barrier during chronic inflammation

**DOI:** 10.1186/s12974-018-1373-4

**Published:** 2018-12-12

**Authors:** Despina Kokona, Andreas Ebneter, Pascal Escher, Martin S. Zinkernagel

**Affiliations:** 1 Department of Ophthalmology, Inselspital, Bern University Hospital, and University of Bern, CH-3010 Bern, Switzerland; 2 Department of Clinical Research, Inselspital, Bern University Hospital, and University of Bern, Bern, Switzerland

**Keywords:** Inflammation, Retina, Microglia, Blood-retina barrier, CSF-1R, Sub-retinal fluid

## Abstract

**Background:**

Microglia-associated inflammation is closely related to the pathogenesis of various retinal diseases such as uveitis and diabetic retinopathy, which are associated with increased vascular permeability. In this study, we investigated the effect of systemic lipopolysaccharide (LPS) exposure to activation and proliferation of retinal microglia /macrophages.

**Methods:**

Balb/c and *Cx3cr1*^*gfp/+*^ mice were challenged with LPS (1 mg/kg) daily for four consecutive days. For microglia depletion, mice were treated with colony-stimulating factor 1 receptor (CSF-1R) inhibitor PLX5622 1 week before the first LPS challenge and until the end of the experiment. In vivo imaging of the retina was performed on days 4 and 7 after the first LPS challenge, using optical coherence tomography and fluorescein angiography. Flow cytometry analysis, retinal whole mount, and retinal sections were used to investigate microglia and macrophage infiltration and proliferation after LPS challenge. Cytokines were analyzed in the blood as well as in the retina. Data analysis was performed using unpaired *t* tests, repeated measures one-way ANOVA, or ordinary one-way ANOVA followed by Tukey’s post hoc analysis. Kruskal-Wallis test followed by Dunn’s multiple comparison tests was used for the analysis of non-normally distributed data.

**Results:**

Repeated LPS challenge led to activation and proliferation of retinal microglia, infiltration of monocyte-derived macrophages into the retina, and breakdown of the blood-retina barrier (BRB) accompanied by accumulation of sub-retinal fluid. Using in vivo imaging, we show that the breakdown of the BRB is highly reproducible but transitory. Acute but not chronic systemic exposure to LPS triggered a robust release of inflammatory mediators in the retina with minimal effects in the blood plasma. Inhibition of the CSF-1R by PLX5622 resulted in depletion of retinal microglia, suppression of cytokine production in the retina, and prevention of BRB breakdown.

**Conclusions:**

These findings suggest that microglia/macrophages play an important role in the pathology of retinal disorders characterized by breakdown of the BRB, and suppression of their activation may be a potential therapeutic target for such retinopathies.

**Electronic supplementary material:**

The online version of this article (10.1186/s12974-018-1373-4) contains supplementary material, which is available to authorized users.

## Background

Endotoxin-induced uveitis (EIU) is an animal model of human uveitis, which can be induced by either a single systemic or a local injection of lipopolysaccharide (LPS). LPS is a component of the outer membranes of gram-negative bacteria and has been shown to bind to Toll-like receptor 4 (TLR4) in iris endothelial cells [1] with subsequently increased inflammatory mediators leading to break down of the blood–ocular barrier, which in turn leads to edema and leukocyte influx [2, 3]. In the retina, expression of TLR4 is upregulated in the inner nuclear layer (INL) and the ganglion cell layer (GCL) after ischemia-reperfusion in rats [4]. Moreover, in vitro studies using rat retinal pigment epithelium (RPE) cells have shown that LPS treatment can lead to a decrease of transepithelial electrical resistance and increased RPE permeability [5]. In the posterior segment of the eye, a single LPS injection leads to increased adhesion of leukocytes in the retinal vascular endothelium and activation of retinal microglia [6].

Microglia are the resident innate immune cells in the central nervous system (CNS). In the retina, they are mainly localized in the inner and outer plexiform layer where they have surveillance function with a highly ramified phenotype [7]. Upon activation, they undergo morphological transformation and adopt an amoeboid phenotype resembling activated macrophages. Microglia, as well as blood inflammatory cells, such as monocytes and neutrophils, play a critical role in the immune system and are commonly activated in response to inflammatory conditions. Microglia/macrophages are activated and increased in number as a result of both the proliferation of resident microglia and recruitment of circulating myeloid cells that infiltrate the retina after injury or infection [8–10]. Microglial over-activation and subsequent inflammation are considered to be implicated in the progression of retinal diseases such as age-related macular degeneration (AMD), or diabetic retinopathy (DR). Specifically, in mouse models of DR, upregulation of inducible nitric oxide synthase (iNOS) has been positively correlated to the disruption of the blood-retina barrier (BRB), since BRB breakdown was prevented in iNOS deficient mice [11–14]. iNOS is mainly expressed by cells such as macrophages, microglia, and astrocytes in response to inflammatory stimuli such as LPS or cytokines [15, 16]. Moreover, microglia/macrophages accumulation in the sub-retinal space has been reported in rodent models of DR [17], while inhibition of microglia’s activation led to a reduction of proinflammatory cytokines production in the diabetic retina [18]. Microglia cells in the diseased retina are able to proliferate [19], secrete proinflammatory cytokines [20, 21], chemokines [22], and reactive oxygen species [23], and they seem to play an important role in retinal disease progression.

In the present study, prolonged systemic inflammation induced by repeated LPS injections led to activation of retinal microglia and infiltration of monocytes, retinal edema, and accumulation of sub-retinal fluid as signs of BRB breakdown. Moreover, a single LPS injection triggered the production of a plethora of inflammatory mediators in the retina and this phenomenon was abolished after repeated injections. In addition, the use of a colony-stimulating factor 1-receptor (CSF1-R) inhibitor, which we have previously shown to dramatically deplete retinal microglia [24], provides evidence that the breakdown of the BRB can be abrogated by suppression of the innate immune response. These data suggest that resident retinal microglia in combination with monocytes invading from the circulation are responsible for the breakdown of the BRB and that BRB integrity can be preserved by inhibition of the CSF-1R.

## Materials and methods

### Animals

Adult (6–8 weeks of age) Balb/c and *Cx3cr1*^*gfp/+*^ mice, hemizygous for the expression of green fluorescent protein (GFP) under the endogenous Cx3c chemokine receptor 1 (Cx3cr1) promoter, on a Balb/c background [25] were used for this study. Animals were housed in groups of 2–5 under temperature and humidity-controlled conditions in individually ventilated cages with a 12-h light/12-h dark cycle. Mice were anesthetized by intraperitoneal injection of 1 mg/kg medetomidine (Dormitor 1 mg/mL, Provet AG, Lyssach, Switzerland) and 80 mg/kg ketamine (Ketalar 50 mg/ml, Parke-Davis, Zurich, Switzerland) for imaging. Atipamezol 2.25 mg/kg (Antisedan 5 mg/mL, Provet AG, Lyssach, Switzerland) was used to antagonize medetomidine after the intervention. At the end of the experiment, mice were euthanized by carbon dioxide (CO_2_) inhalation.

### Systemic LPS challenge

Mice were intravenously injected (tail vein) with 150 μl lipopolysaccharides (LPS) from *Escherichia coli* [1 mg/kg in 1x phosphate-buffered saline (PBS), pH 7.4; Sigma-Aldrich] for four consecutive days. Control mice received intravenous injections of PBS instead of LPS. Retinal imaging was performed at baseline (before LPS injection), day 4 (approximately 6 h after the last LPS challenge) and day 7 (3 days after the last LPS challenge). One group of mice (*n* = 4 Balb/c and *n* = 8 *Cx3cr1*^*gfp/+*^ mice) was euthanized at day 4, and a second group (*n* = 8 *Cx3cr1*^*gfp/+*^ mice) was euthanized at day 7 immediately after the imaging session.

### Inhibition of colony-stimulating factor-1 receptor (CSF-1R) with PLX5622

The CSF-1R inhibitor PLX5622 was incorporated into the mouse chow (1200 ppm formulated in AIN-76A standard rodent diet; Research Diets, Inc., New Brunswick, NJ, USA, provided by Plexxikon, Inc.). One group of *Cx3cr1*^*gfp/+*^ mice (*n* = 6) had ad libitum access to PLX5622-chow 1 week before the LPS challenge and until the end of the experiment, while a second group of animals (*n* = 6) was fed with control chow (provided by Plexxikon, Inc.). Microglia depletion by PLX5622 was evaluated 1 week after the initiation of PLX5622 treatment with autofluorescent imaging. LPS (1 mg/kg in PBS) was administered intravenously immediately after the imaging session for four consecutive days, and mice were imaged approximately 6 h after the last LPS challenge and euthanized immediately after.

### Imaging

Before and after the LPS challenge, mice were imaged with spectral domain optical coherence tomography (SD-OCT) for the evaluation of retinal structure, autofluorescence imaging (AF) for the visualization of GFP-positive cells, and fluorescein angiography (FA) for the visualization of retinal vasculature using a Heidelberg Spectralis device (Heidelberg Engineering GmbH, Heidelberg, Germany). Mice were anesthetized and placed on a custom-made platform positioned on the chin rest of the SD-OCT device. No contact lens was used during image acquisition. Pupils were dilated with a drop of tropicamide 0.5%/phenylephrine 2.5% (Hospital Pharmacy, Inselspital, Bern, Switzerland), and hydroxypropylmethylcellulose (Methocel 2%; OmniVision, Neuhausen, Switzerland) was applied to each eye during imaging to keep the cornea hydrated. The infrared mode was used to focus on retinal vessels and AF images were acquired with a 102° lens at high resolution of 1536 × 1636 pixels. After examination of both eyes, SD-OCT was performed using a 55° lens at a high resolution of 1008 × 596 pixels in grid mode. In total, 43 to 61 images were acquired centered on the optic nerve head. For fluorescein angiography, 50 μl of fluorescein solution (Fluorescein 10% Faure; Novartis, Switzerland; 0.01% in PBS) were injected subcutaneously and images were acquired with a 55° or a 102° lens to visualize the vascular plexus.

### Retinal whole mounts

At the end of the experiments, mice were euthanized with CO_2_ inhalation and their eyes were removed. From each mouse, one eye was used for retinal whole mounts and the other one was used to obtain vertical retinal sections (see below). For retinal whole mounts, the eyes were processed as reported elsewhere [26]. Retinas were incubated overnight at 4 °C with isolectin IB4 from *Griffonia simplicifolia* (GS-IB4; Alexa Fluor 647 conjugate; 1:100, ThermoFisher Scientific, Waltham, MA, USA) for labeling of retinal vasculature, a rabbit polyclonal antibody against ionized calcium-binding adapter molecule 1 (anti-iba1 rabbit polyclonal antibody, 1:500, Wako Cat #019-19741, Wako Pure Chemical Industries Ltd., Osaka, Japan) for labeling of microglia/macrophages, and/or a chicken polyclonal antibody against GFP (1:100, ab13970, Abcam, Cambridge, UK) for the labeling of microglia/macrophages in *Cx3cr1*^*gfp/+*^ mice. Retinas were washed with 1x PBS 0.2% Triton X-100 (Sigma-Aldrich, St. Louis, MO, USA) and incubated with the secondary antibodies goat anti-rabbit IgG (H&L) Alexa Fluor 594 conjugate (1:1000, A27016, ThermoFisher scientific, Waltham, Massachusetts, USA) for Iba-1 and/or a pre-absorbed goat polyclonal antibody to chicken IgY H&L (FITCH) (1:1000, ab7114, Abcam, Cambridge, UK) for GFP. Retinas were then washed in 1x PBS 0.2% Triton X-100; four radial cuts were made and tissues were flat-mounted on a slide having the ganglion cell layer facing up. Whole mounts were cover-slipped and examined under a confocal microscope. Primary and secondary antibodies were diluted in 1x PBS, 0.2% Triton X-100, 5% normal goat serum (NGS, X0907, DAKO AG, Wiesentheid, Germany).

### Immunohistochemical studies

One eye from each euthanized mouse was fixed in 4% paraformaldehyde (PFA, pH 7.4) for 24 h. Eyes were processed for routine paraffin embedding, and slices of 5 μm were cut with a microtome (Leica Biosystems, Muttenz, Switzerland) from the central retina including the optic nerve head. Slides were deparaffinized, rehydrated, and endogenous peroxidase activity was blocked by incubation with 0.5% H_2_O_2_ in methanol. Antigen retrieval was achieved by incubation in citrate buffer (10 mM, pH 6.0, 95 °C, 10 min). Slides were blocked for 30 min with 5% normal goat serum (NGS) in 0.01% Triton X-100 and incubated with the anti-Iba-1 antibody (see above) overnight at 4 °C. Slides were washed with PBS and incubated with a biotinylated goat anti-rabbit IgG antibody (1:1000; Vector labs, Burlingame, CA) for 60 min at room temperature. Slides were washed again and incubated with Horseradish Peroxidase-Streptavidin (1:1000; Vector labs, Burlingame, CA), for 60 min at room temperature. Slides were treated with the NovaRED Peroxidase (HRP) Substrate Kit (Vector labs, Burlingame, CA) according to the manufacturer instructions, washed in distilled water, counterstained with hematoxylin and mounted in non-aqueous mounting medium (Eukitt; O. Kindler GmbH & Co, Freiburg, Germany).

For the labeling of neutrophils and aquaporin 4 (AQP4), slides were treated as above, and after the antigen retrieval, they were blocked for 30 min with 1x PBS, 5% NGS, 0.1% Triton X-100 and incubated overnight at 4 °C with a rabbit monoclonal antibody against myeloperoxidase (MPO, 1:1000, ab208670, Abcam, Cambridge, UK) or a rabbit polyclonal antibody against AQP4 (1:500, AQP-014, Alomone labs, Jerusalem, Israel). For the investigation of microglia proliferation in *Cx3cr1*^*gfp/+*^ mouse retinas, a rabbit polyclonal antibody against Ki67 (1:1000, Abcam, Cambridge, UK) was used in combination with the chicken anti-GFP (see above). Slides were mounted with mounting medium containing DAPI (Vector Laboratories, Burlingame, CA, USA), cover-slipped, and examined under the microscope. The secondary antibodies goat anti-rabbit IgG (H&L), Alexa Fluor 594 conjugate (1:1000, A27016, ThermoFisher scientific, Waltham, Massachusetts, USA) and Alexa Fluor 488 conjugate (1:1000, A11008, ThermoFisher scientific, Waltham, Massachusetts, USA) were used for the visualization of Ki67, and MPO or AQP4 staining, respectively.

### Microscopy

An epifluorescence microscope (Olympus BX60 microscope; Olympus, Tokyo, Japan) was used for the examination of retinal sections. Retinal whole mounts were imaged using an inverted Zeiss LSM 710 fluorescence confocal microscope (Carl Zeiss, Oberkochen, Germany). Z-stacks of 100.8 ± 15.1 μm (mean ± standard deviation [SD]) with 5-μm interval were obtained and analyzed with the ZEN system 2011 software (Carl Zeiss). Photoshop (Version 7.0, Adobe Systems, San Jose, CA, USA) was used to adjust light and contrast. All final figures were prepared in CorelDraw (Corel Corporation of Ottawa, Canada).

### Microglia quantification and retinal thickness measurements

Retinal microglia (GFP^+^ cells) was quantified on the whole area of each single AF image using the ImageJ software [27] and the “Analyze particles” function as reported elsewhere [28]. For the estimation of microglia occupied area, color threshold was adjusted in retinal AF images and the pixel areas of the binary mask was calculated and expressed as a percentage of the total pixel area. Retinal thickness in OCT volume scans was measured in the four inner Early Treatment Diabetic Retinopathy Study (ETDRS) subfields, using the Orion™ software (Voxeleron LLC, Pleasanton, CA, USA). Correction of the lines marking the Bruch’s membrane and the inner limiting membrane were manually performed before thickness measurements.

### Protein extraction

In total, 36 Balb/c mice were used for protein extraction and subsequent analysis of protein levels in retinas and blood plasma using a mouse inflammation antibody array-membrane kit (Abcam, Cambridge, UK). One group of mice (*n* = 12) had ad libitum access to PLX5622 chow for 1 week before the LPS challenge and until the end of the experiment. A second group of mice (*n* = 24) was fed with control chow for the whole duration of the experiment. PLX5622-fed and control mice were challenged with LPS (1 mg/kg) and euthanized 12 or 24 h after the first LPS injection or 6 h after the fourth daily LPS injection. For protein extraction, the cornea and the lens were removed, the retina was mechanically detached from the remaining eyecup and placed in lysis buffer [1x lysis buffer provided with the kit, 1 mM Na_3_VO_4,_ protease inhibitor cocktail (Roche, Basel, CH)]. Tissue homogenization was performed with a Precellys 24 tissue homogenizer (Bertin Instruments, Montigny-le-Bretonneux, France). Four retinas were pooled as one sample. Homogenized tissues were incubated for 30 min on ice before being centrifuged for 20 min at 13000 rpm at 4 °C. The supernatant was transferred into a clean tube, and total protein concentration was determined using the Bradford assay.

### Blood plasma preparation

Peripheral blood was collected from mice used for protein extraction (see above). Briefly, mice were intraperitoneally injected with a lethal dose of pentobarbital (≥ 200 mg/kg; Esconarkon, Streuli Pharma AG, Uznach, 300 mg/ml) and blood was collected by cardiac puncture, in tubes containing ethylenediaminetetraacetic acid (EDTA) (pH 8, 0.5 M), to prevent blood coagulation. Blood samples were gently mixed and centrifuged for 10 min, at 2000 g and at 4 °C. The supernatant was collected in new tubes and 5 μl of a 2x stock protease inhibitors cocktail (cOmplete ULTRA Tablets, EDTA-free; Roche, Basel, CH) was added per 100 μl of plasma. Total protein concentration of plasma samples was determined using the Bradford assay.

### Mouse inflammation antibody array-membranes

Antibody array-membranes (Abcam, Cambridge, UK) were processed according to manufacturer’s instructions. Briefly, the membranes were blocked for 2 h in blocking buffer (provided with the kit) and incubated with 500 μg of total protein overnight at 4 °C. Membranes were extensively washed with wash buffer (provided with the kit) and were incubated with 1 ml of biotin-conjugated anti-cytokines (provided with the kit) in blocking buffer overnight at 4 °C with gentle shaking. Membranes were washed again and incubated with 1x HRP-conjugated streptavidin (provided with the kit) for 2 h at room temperature. Chemiluminescence detection was performed using detection buffers provided with the kit and a Fusion Pulse Imaging System (Witec AG, Luzern, CH). Densitometry analysis was performed using the “Protein Array Analyzer” function of the ImageJ software [27]. The background was subtracted, and the signal was normalized between different membranes using the positive control spots.

### Flow cytometry analysis

*Cx3cr1*^*gfp+/−*^ mice treated with either four daily doses of LPS (1 mg/kg) or equal volume of PBS were euthanized approximately 6 h after the last LPS challenge, and their retinas were used for flow cytometry analysis. Both retinas of each mouse were analyzed as one sample. Retinas were processed according to Ebneter et al. [8]. Before antibody staining, single cells suspensions were incubated with Zombie Red™ Fixable Viability Kit (Biolegend, 1:800) in Dulbecco’s phosphate-buffered saline (DPBS^−/−^, ThermoFisher Scientific)/0.01% DNase I (Roche), for detection of dead cells, according to the manufacturer’s instructions. For antibody staining, the samples were washed, re-suspended in flow cytometry buffer (5 mM EDTA, 20% FBS, 0.5% Na azide, in DPBS^−/−^/0.01% DNase I) and incubated for 20 min with an Fc blocker (Biolegend, 1:200). Microglia and macrophages were subsequently stained with fluorescent-labeled antibodies against CD45 (30-F11, 1:400), CD11b (M1/70, 1:200) and MHC-II (major histocompatibility complex class-II, AF6-120.1, 1:200), for 20 min at 4 °C in the dark. Samples were washed again and re-suspended in 0.1% PFA (pH 7.4) for 10 min at 4 °C in the dark. The samples were washed two times, re-suspended in flow cytometry buffer, and analyzed. All washing steps involved addition of 1 ml DPBS^−/−^/0.01% DNase on each sample and centrifugation at 500 g for 3 min, at 4 °C.

Data were acquired with an LSR II Cytometer System and the BD FACSDiva software (BD Biosciences, Allschwil, Switzerland). The data were analyzed with the Flowjo Single Cell Analysis Software V10 (TreeStar, Ashland, OR, USA). All antibodies were purchased from Biolegend (San Diego, CA, USA).

### Statistics

GraphPad Prism 5.0 software (GraphPad Software, Inc., San Diego, CA, USA) was used for the statistical analysis. The normal distribution of the different data sets was evaluated using the Shapiro-Wilk normality test. Statistically significant differences of GFP-positive cell numbers or areas occupied by GFP-positive cells on AF images were determined using repeated measures one-way ANOVA followed by Tukey’s post hoc analysis. For the comparison of the flow cytometry data, unpaired *t* tests were used. Retinal thickness measurements were compared using ordinary one-way ANOVA followed by Tukey’s post hoc analysis. For the comparison of cytokine/chemokine expression levels, ordinary one-way ANOVA followed by Tukey’s post hoc analysis was used for normally distributed data and Kruskal-Wallis test followed by Dunn’s multiple comparison test was used for data following non normal distribution. *P* values < 0.05 were considered statistically significant. The quantification data are presented as mean ± SD throughout the manuscript and figures.

## Results

### Effect of systemic LPS treatment on blood-retina barrier integrity

**Fig. 1:**
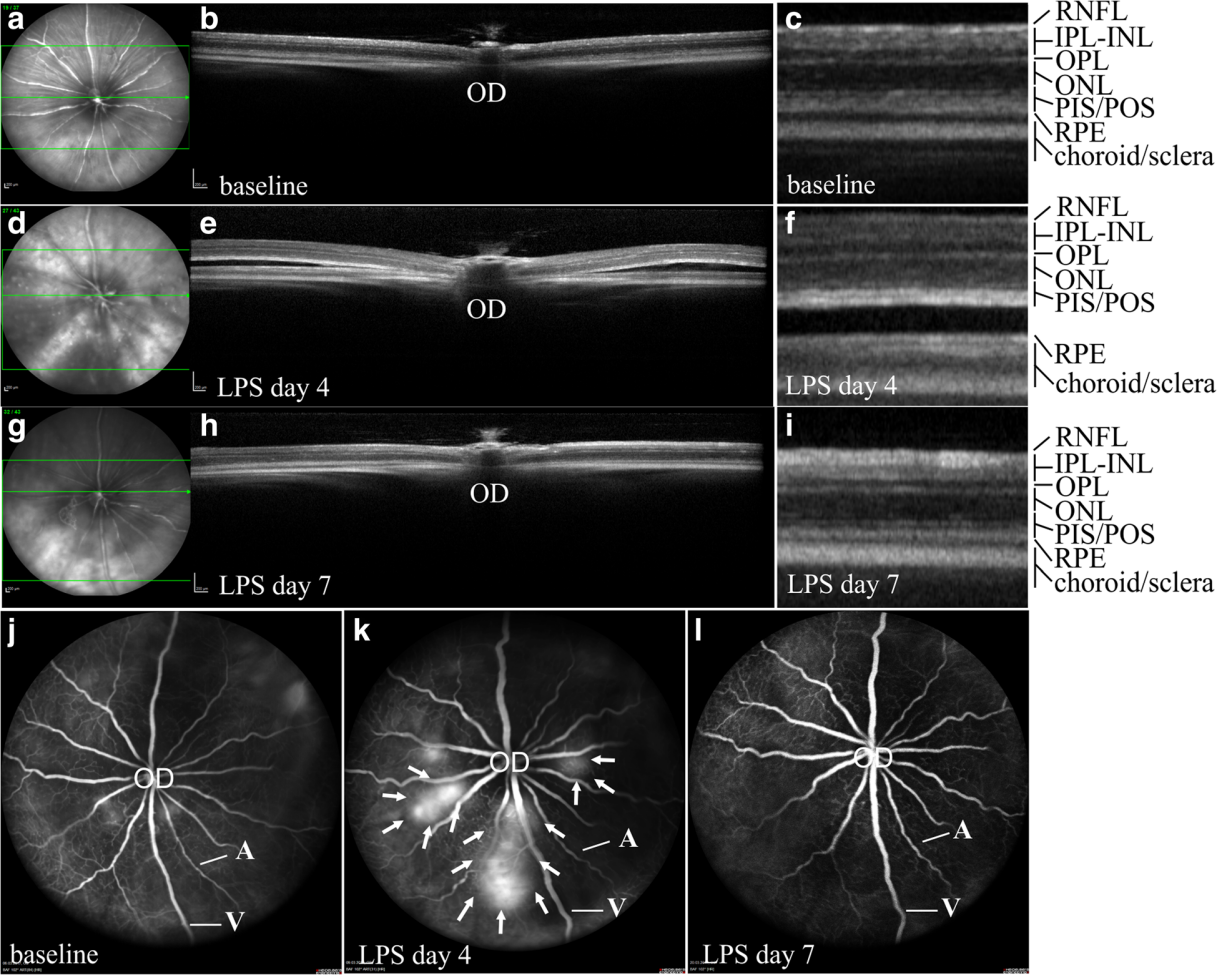
Disruption of the blood-retina barrier in LPS-challenged mice. Infrared images (**a**, **d**, **g**) and vertical B-scans (**b**, **e**, **h**), at baseline (**a**, **b**), at day 4 of the LPS challenge (**d**, **e**), and at day 7 after the first LPS challenge (**g**, **h**) in Balb/c mice, revealed accumulation of sub-retinal fluid after 4 daily systemic injections of LPS. At higher magnification individual retinal layers can be seen at baseline (**c**), at day 4 of the LPS challenge (**f**), and at day 7 after the first LPS challenge (**i**). Sub-retinal fluid was accumulated in LPS-treated mice at day 4 (**f**). Fluorescein angiograms at baseline (**j**), at day 4 of the LPS challenge (**k**), and at day 7 after the first LPS challenge (**l**) revealed fluorescein leakage in LPS-treated mice at day 4 (**k**). Arrows correspond to the leakage area. Location of B-scans in **b**, **e**, and **h** is indicated with the middle line in **a**, **d**, **g**, respectively. One representative retina out of ≥ 8 is presented for each time point. A, artery; INL, inner nuclear layer; IPL, inner plexiform layer; OD, optic disk; ONL, outer nuclear layer; OPL, outer plexiform layer; PIS/POS, photoreceptor inner segments/photoreceptor outer segments; RNFL, retinal nerve fiber layer; RPE, retinal pigment epithelium; V, vein

In vivo imaging of Balb/c mice with SD-OCT and FA, before and after four daily injections of LPS as well as 7 days after the first LPS challenge (Fig. 1), revealed a normal retinal structure and retinal vasculature at baseline (Fig. 1a–c) and accumulation of sub-retinal fluid at day 4 of LPS challenge (Fig. 1d–f). At day 7, retinal structure was comparable to the baseline (Fig. 1g–i). Leakage of fluorescein was observed in fluorescein angiograms of LPS-challenged mice at day 4 (Fig. 1k), suggesting disruption of the BRB, but not at day 7 (Fig. 1l).

### Effect of systemic LPS challenge on microglia/macrophages population in the retina

**Fig. 2:**
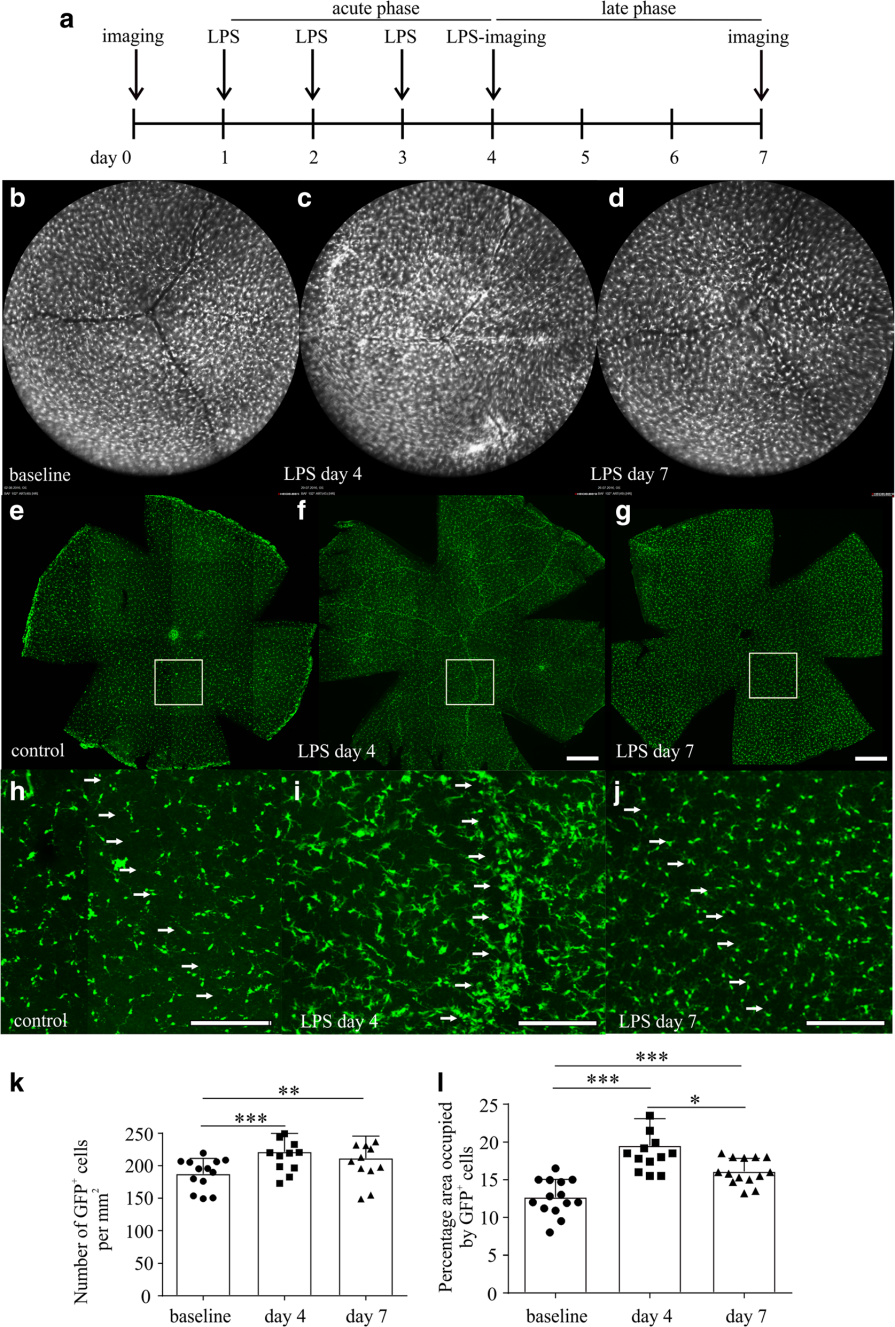
Accumulation of microglia/macrophages around retinal vessels in LPS-challenged mice. Experimental setup (**a**) and representative autofluorescence images of microglia/macrophages in the retina at baseline (**b**), after the fourth LPS injection (**c**) and 3 days after the last LPS injection (**d**). GFP-positive cells were identified as white signal on the autofluorescence images. One representative retina out of ≥ 8 is presented for each time point. Retinal whole mounts from untreated (**e**, control) and LPS-treated *Cx3cr1*^*gfp/+*^ mice stained with GFP at day 4 (**f**) and at day 7 (**g**) after the first LPS injection. One representative retina out of ≥ 4 is presented for each time point. Higher magnification of the delineated areas in **e**, **f**, and **g** are depicted in **h**, **i**, and **j**, respectively. The numbers of GFP-positive cells were elevated in the retina at day 4 of the LPS challenge and 3 days after the last LPS challenge, compared to the baseline (**k**; **p < 0.01, ***p < 0.0001; *n* = 13 eyes for each time point; repeated measures one-way ANOVA followed by Tukey’s post hoc analysis). The area occupied by microglia cells in the retina was elevated after the LPS challenge and was decreased 3 days after the termination of the inflammatory stimulus (**l**; *n* = 14 eyes for each time point; *p < 0.05, **p < 0.01, ***p < 0.001; repeated measures one-way ANOVA followed by Tukey’s post hoc analysis). Arrows represent the location of retinal vessels. Scale bars: 500 μm (**e**, **f**, **g**); 200 μm (**h**, **i**, **j**)

**Fig. 3:**
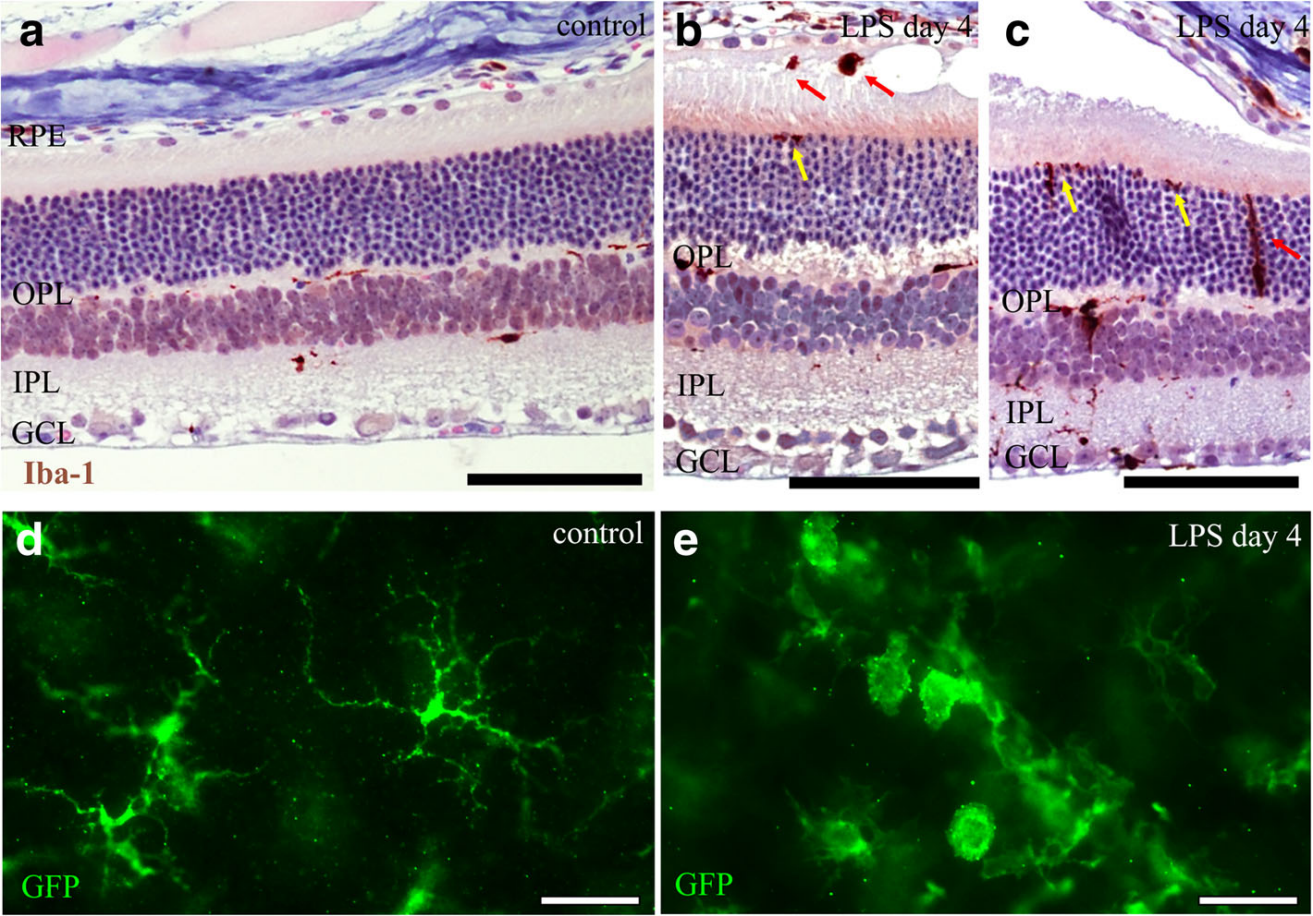
Activation and migration of retinal microglia/macrophages after LPS challenge in *Cx3cr1*^*gfp/+*^ mice. In control retinas (**a**), Iba-1-positive cells were mainly found in the inner and outer plexiform layer (IPL and OPL, respectively). In LPS-challenged retinas (**b**, **c**), Iba-1-positive microglia/macrophages were also detected in the proximity of the sub-retinal fluid accumulation (red arrows in **b**) and in the distal ONL (yellow arrows in **b** and **c**). Iba-1-positive cells migrating towards the outer retina were also detected (red arrow in **c**). In control retinal whole mounts, GFP-positive microglia cells have a ramified morphology typical for resting microglia (**d**). After LPS challenge, activated GFP-positive cells with retracted processes and round cytosol were detected in the retina (**e**). One representative retina out of ≥ 4 is presented for each time point. Scale bars: 100 μm. GCL, ganglion cell layer; IPL, inner plexiform layer; OPL, outer plexiform layer; RPE, retinal pigment epithelium

Microglia/macrophage populations in the retinas of *Cx3cr1*^*gfp+/−*^ mice were monitored in vivo with AF imaging before and after the LPS challenge (Fig. 2a). At baseline, the GFP^+^ cells were normally distributed (Fig. 2b), while accumulation of microglia/macrophages around retinal vessels was observed at day 4 of systemic LPS challenge (Fig. 2c). This was reversed 3 days after the last LPS injection (Fig. 2d). Retinal whole mounts stained with GFP substantiated this observation (Fig. 2e–j). The number of GFP-positive cells was increased by 18% at day 4 of LPS challenge (Fig. 2k; 221.3 ± 28.66 cells per mm^2^ at day 4 compared to 187.5 ± 23.83 cells per mm^2^ counted at baseline, ***p < 0.001, *n* ≥ 8 retinas per time point) and remained elevated 3 days after the last LPS challenge (Fig. 2k; 211.6 ± 34.23 cells per mm^2^, **p < 0.01; *n* ≥ 8 retinas). Microglia/macrophages occupied a larger retinal area at day 4 of LPS challenge (Fig. 2l; ***p < 0.001, *n* ≥ 8 retinas), which decreased 3 days later (Fig. 2 l; *p < 0.05, *n* ≥ 8 retinas) while still increased compared to baseline (Fig. 2l; ***p < 0.001, *n* ≥ 8 retinas). Iba-1-positive microglia/macrophages were mainly found in the inner and outer plexiform layer of the control retina (Fig. 3a). Four days after repetitive LPS challenges, Iba-1-positive cells could be also detected in the sub-retinal space (Fig. 3b; red arrows) and in the distal ONL (Fig. 3b, c; yellow arrows). Iba-1-positive cells migrating from the inner retina towards the ONL were also detected (Fig. 3c; red arrow). Examination of retinal whole mounts by confocal microscopy revealed a ramified morphology of GFP-positive cells in the control retinas (Fig. 3d), while in LPS-challenged-mice an amoebic-like morphology as signs of activation was observed (Fig. 3e).

### Quantification of microglia/macrophages with flow cytometry

**Fig. 4:**
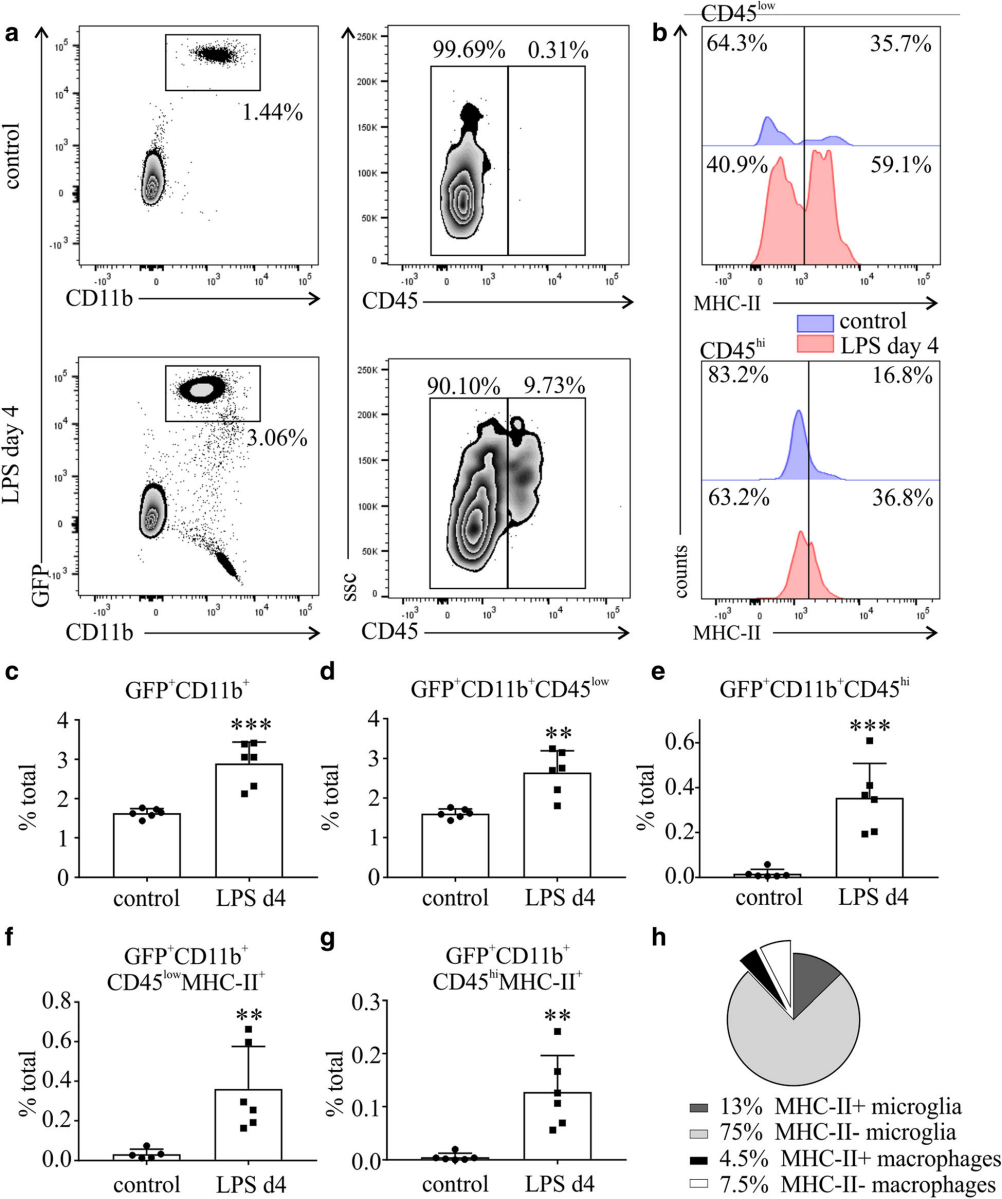
Flow cytometry analysis of different microglia/macrophages populations in the retina after systemic LPS challenge. Microglia/macrophages were gated as GFP^+^CD11b^+^ cells (**a**, left panel) and were further gated as CD45^low^ microglia and CD45^hi^ macrophages (**a**, right panel). MHC-II expression in microglia and macrophages was increased in the LPS-challenged group (**b**). The numbers of GFP^+^CD11b^+^ microglia/macrophages (**c**, ***p < 0.001, unpaired *t* test), GFP^+^CD11b^+^CD45^low^ microglia (**d**, **p < 0.01, unpaired *t* test) and GFP^+^CD11b^+^CD45^hi^ macrophages (**e**, ***p < 0.001, unpaired *t* test) were elevated in the LPS group. Microglia (**f**, **p < 0.01, unpaired *t* test) and macrophages (**g**, **p < 0.01, unpaired *t* test) activation was also increased in the LPS group. The pie chart depicts the composition of the GFP^+^ cells in the retina after four consecutive LPS challenges (**h**)

Flow cytometry was performed on the retinas of *Cx3cr1*^*gfp+/−*^ mice treated with either LPS (1 mg/kg) or PBS for four consecutive days, approximately 6 h after the last LPS injection. After selection of single-live cells, the GFP^+^CD11b^+^ population has been selected and further gated as CD45^low^ (microglia) and CD45^hi^ (macrophages) (Fig. 4a). A population of GFP^−^CD11b^+^ cells was detected in the LPS group, most probably representing neutrophils and/or dendritic cells (Fig. 4a, bottom left panel). A few GFP^+^CD11b^+^ macrophages were present in the control retinas, while their number was elevated after 4 LPS challenges (Fig. 4a, right top and bottom panels). A higher number of microglia and macrophages expressed the activation marker MHC-II after the LPS challenge compared to the control retinas (Fig. 4b). Quantification of different cell populations revealed elevated numbers of GFP^+^CD11b^+^, GFP^+^CD11b^+^CD45^low^, and GFP^+^CD11b^+^CD45^hi^ cells in the retinas of the LPS-challenged mice (Fig. 4c–e, respectively; **p < 0.01, ***p < 0.001 compared to control; unpaired *t* test). MHC-II was used as an activation marker and the numbers of GFP^+^CD11b^+^CD45^low^MHC-II^+^ microglia and GFP^+^CD11b^+^CD45^hi^MHC-II^+^ macrophages were quantified. Statistically significant increase of microglia and macrophages activation was observed in the LPS group (Fig. 4f, g, respectively; **p < 0.01 compared to control; unpaired *t* test). The majority of GFP^+^CD11b^+^ cells in the LPS retina corresponded to microglia and 14.8% of them (13% of total GFP^+^CD11b^+^ cells) expressed the activation marker MHC-II. Macrophages represented almost 12% of the total GFP^+^CD11b^+^ population and about 37.5% of them (4.5% of total GFP^+^CD11b^+^ cells) were MHC-II^+^ (Fig. 4h).

### Effect of the CSF-1 receptor inhibitor PLX5622 on microglia population in the retina

**Fig. 5:**
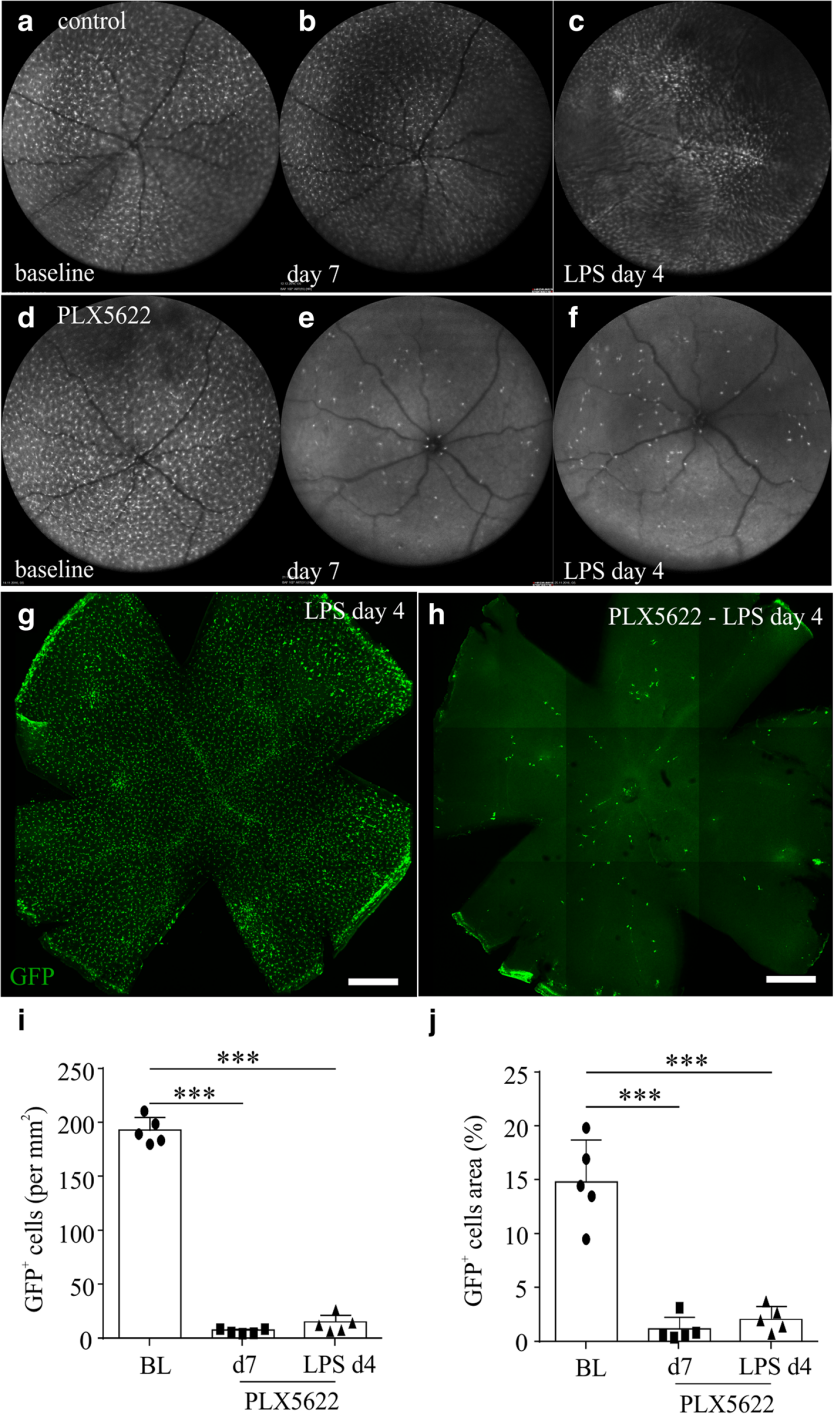
Depletion of retinal microglia using a CSF-1R tyrosine kinase inhibitor (PLX5622). *Cx3cr1*^*gfp/+*^ mice were imaged at baseline, were fed with control chow or PLX5622 chow for the rest of the experiment, and were injected with LPS at day 7 after the initiation of the diet. LPS was injected intravenously for four consecutive days and retinas were analyzed after the last LPS injection. Representative autofluorescence images of LPS-challenged *Cx3cr1*^*gfp/+*^ mice fed with control or PLX5622 chow at baseline (**a**, **d**, respectively), 7 days after the initiation of the diet (**b**, **e**, respectively) and after 4 daily LPS injections (**c**, **f**, respectively). In retinal whole mounts of LPS-challenged mice fed with control chow, GFP-positive cells were observed throughout the retina and were accumulated around retinal vessels (**g**). Incorporation of PLX5622 into the mouse chow led to a reduction of GFP-positive cells in the retina at day 4 of the LPS challenge (**h**). One representative retina out of ≥ 8 is presented for each time point. Counting of GFP-positive cells on autofluorescence images showed dramatic reduction of the number of GFP-positive cells (**i**, *n* = 5 mice; ***p < 0.001; repeated measures one-way ANOVA followed by Tukey’s post hoc analysis), as well as the area they occupied (**j**, *n* = 6 mice; ***p < 0.001; repeated measures one-way ANOVA followed by Tukey’s post hoc analysis), after PLX5622 treatment. Scale bars: 500 μm

**Fig. 6:**
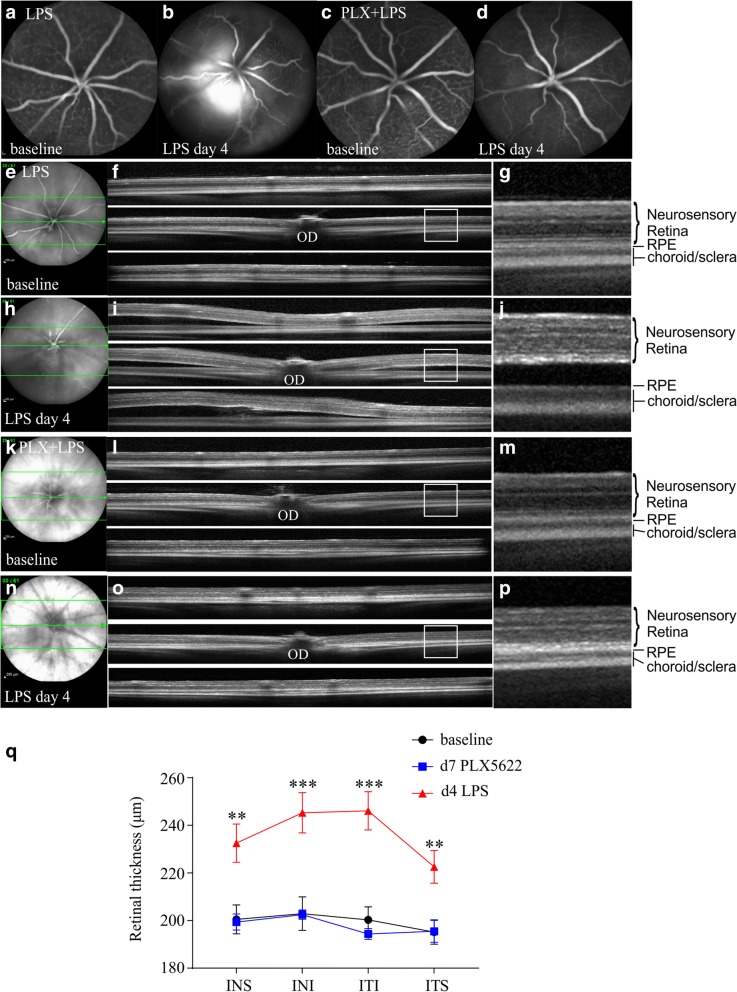
Inhibition of LPS-induced BRB disruption and retinal swelling using a CSF-1R tyrosine kinase inhibitor (PLX5622). Representative fluorescein angiograms of LPS-challenged *Cx3cr1*^*gfp/+*^ mice fed with control chow (**a**, **b**) or PLX5622 chow (**c**, **d**), before (**a**, **c** for control and PLX5622, respectively) and after four daily injections of LPS (**b**, **d** for control and PLX5622, respectively). In mice fed with the PLX5622 chow, no fluorescein leakage is observed at day 4 of the LPS challenge (**d**). Vertical B-scans of mouse retinas fed with control (**e–j**) or PLX5622 chow (**k–p**) at baseline (**e–g** and **k–m** for control and PLX5622, respectively) and after the fourth LPS injection (**h–j** and **n–p** for control and PLX5622, respectively). The areas of the B-scans are indicated with lines in the infrared images. Magnifications of the areas inside the white boxes are presented in **g**, **j**, **m**, and **p**. After 4 days of LPS injections, accumulation of sub-retinal fluid was present in the retinas of control-fed mice (**h**–**j**), while PLX5622-fed mice had a normal retinal structure with no accumulation of sub-retinal fluid (**n–p**). One representative retina out of ≥ 8 is presented for each time point. Total retinal thickness was increased at day 4 of the LPS challenge compared to the baseline (**q**, **p < 0.01, ***p < 0.001, *n* = 14 eyes for each time point; ordinary one-way ANOVA followed by Tukey’s post hoc analysis), while PLX5622 prevented retinal swelling

In vivo AF imaging and staining of retinal whole mounts with GFP, in *Cx3cr1*^*gfp+/−*^ mice fed with the CSF-1R inhibitor PLX5622, revealed dramatic differences in the number of GFP-positive cells before and after the initiation of PLX5622 diet (Fig. 5d, e). The number of GFP-positive cells was diminished by approximately 97% after 7 days of PLX5622 diet (Fig. 5e, i, ***p < 0.001; BL, 189.2 ± 12.4 cells per mm^2^; PLX5622 day 7, 5.8 ± 2.0 cells per mm^2^, *n* ≥ 8 retinas per time point). Furthermore, the area occupied by microglia was significantly reduced by PLX5622 compared to baseline (Fig. 5j). Challenging of PLX5622-fed mice with LPS did not yield any statistically significant increase in the number of GFP-positive cells or the occupied area (Fig. 5f, h, i, j, 12.9 ± 7.9 cells per mm^2^, *n* ≥ 8 retinas per time point). In all mice fed with PLX5622 throughout the experiment, no fluorescein leakage (Fig. 6c, d) or accumulation of sub-retinal fluid (Fig. 6k-p) was observed. Moreover, retinal swelling that was observed after four daily LPS injections was totally abrogated by PLX5622 (Fig. 6q, **p < 0.01, ***p < 0.001, *n* ≥ 7 retinas).

### Effect of systemic LPS challenge on inflammatory cytokine and chemokines levels in the retina

The relative expression of inflammatory mediator levels, which were significantly changed compared to control retinas, is summarized in Table 1. A pronounced increase of more than 20 fold was detected in several chemokines (e.g., CCL3, CCL12, CRG-2), proinflammatory cytokines (e.g., IL-6, IL-9, IL-17) and growth factors (e.g., IGFBP-6, GM-CSF), 24 h after the first systemic LPS insult. Less but still pronounced increase was observed in several other pro- (e.g., IL-1α, IL-12p70, IL-3, TNF-α) and anti-inflammatory cytokines (e.g., IL-13, IL-4) 24 h after a single LPS challenge. Repetitive LPS challenge (4 daily injections) abolished the increase in most of cytokine levels in the retina, while it did lead to an increase of basic fibroblast growth factor (bFGF). PLX5622 abolished the LPS-induced increase on cytokines expression with only few exceptions (e.g., CXCL1, I-CAM, bFGF).

**Table 1:** Effect of LPS and LPS + PLX5622 in cytokine levels in the mouse retina

	24 h		96 h	
	LPS	LPS + PLX5622	LPS	LPS + PLX5622
Chemokines				
CCL2	19.1***	1.46^###^	1.13	1.24***^#^
CCL3	22***	1.32^###^	0.88	0.85
CCL5	2.32***	0.48^###^	0.84	0.85
CCL9	2.51**	0.59^###^	0.98	0.97
CCL11	7.73***	0.55^###^	0.90	0.92
CCL12	21.65***	0.76^###^	1.05	0.99
CCL17	20.07***	0.94^###^	0.94	1.12
CCL19	10.49***	0.69^###^	0.94	1.05
CCL20	12.63***	1.16^###^	0.83	1.03
CCL24	8.15***	0.60^###^	1.05	1.0
CCL27	10.70***	0.67^###^	0.92	1.07
CRG-2	35.83***	1.34^###^	0.73	0.96
CX3CL1	17.89***	0.84^###^	0.85	0.83
CXCL1	6.34***	3.16*^###^	1.06	1.10
CXCL2	8.61***	0.78^###^	1.04	1.12
CXCL4	9.81***	0.73^###^	1.07	1.10
CXCL5	8.16***	0.99^###^	0.96	1.03
CXCL9	10.51***	0.66^###^	0.81	0.83
CXCL12	15.65***	0.92^###^	0.85	1.02
CXCL13	13.3***	0.76^###^	0.86	0.96
CXCL16	7.89***	0.72^###^	0.97	1.02
XCL1	14.74***	1.09^###^	1.09	1.11
Growth factors				
bFGF	0.87	1.16^###^	3.38*	1.33^#^
IGFBP-3	11.17***	0.77^###^	0.91	1.11
IGFBP-5	8.79***	0.64^###^	0.75	0.77
IGFBP-6	24.18***	1.28^###^	0.86	0.90
G-CSF	11.25***	2.45^###^	0.74	0.78
GM-CSF	25.42***	1.04^###^	0.87	0.95
M-CSF	10.22***	0.8^###^	1.05	1.17
VEGF	10.73***	0.68^###^	0.78	0.8
TNF superfamily				
CD30	40.00***	2.60^###^	0.83	0.88
CD30 L	23.08***	1.10^###^	0.70	0.85
CD40	67.50***	3.25^###^	0.94	1.08
Fas ligand	5.48***	0.59^###^	0.71	0.76
sTNFRI	5.06***	0.91^###^	0.89	0.93
sTNFRII	8.39***	0.86^###^	0.99	0.96
TNF-α	14.46***	0.77^###^	1.65	0.74
cell adhesion molecules				
I-CAM	1.40	3.29***^###^	1.04	1.27
VCAM-1	1.56*	0.62^###^	1.58**	1.42
L-selectin	7.77***	0.72^###^	0.81	0.91
P-selectin	12.67***	0.92^###^	0.94	1.00
Proinflammatory cytokines				
IFN-γ	21.94***	1.25^###^	0.81	0.86
IL-1α	9.05***	0.73^###^	0.99	0.56*^#^
IL-1β	16.73***	0.8^###^	0.85	0.98
IL-3	11.76***	0.3^###^	0.78	0.75
IL-3 Rb	11.62***	0.59^###^	0.92	0.8
IL-6	25.67***	1.22^###^	0.84	0.83
IL-9	27.91***	1.17^###^	0.85	0.82
IL-12 p70	16.26***	0.78^###^	1.00	1.12
IL-17	29.23***	1.13^###^	0.90	1.06
Leptin	14.70***	0.5^###^	0.85	1.04
Leptin R	20.01***	0.67^###^	0.96	1.02
TCA-3	10.25***	0.72^###^	1.12	1.12
TIMP-1α	3.07***	0.70^###^	0.94	0.86
Anti-inflammatory cytokines				
IL-4	17.36***	0.73^###^	1.21	1.22
IL-5	28.83***	1.56^###^	0.79	0.83
IL-10	42***	1.05^###^	1.08	1.14
IL-13	19.16***	0.88^###^	0.90	0.95

All data are normalized to control retinas. *p < 0.05, **p < 0.01, ***p < 0.001 compared to control; ^#^*p* < 0.05, ^###^*p* < 0.001 compared to LPS

### Effect of systemic LPS challenge on inflammatory cytokine and chemokines levels in the peripheral blood plasma

The expression of 23 cytokines and chemokines was examined in the peripheral blood plasma from LPS-challenged mice in the absence or presence of PLX5622 (Table 2). Twelve [10] hours after a single LPS challenge only granulocyte colony-stimulating factor (G-CSF) levels were elevated in the blood plasma and remained elevated at 24 h and after 4 daily LPS injections (96 h). At 24 h, an increase on MMP-3 protein levels was detected, and after repeated LPS exposures, CXCL1, G-CSF, IL-4, and MMP-3 were all increased compared to control. The presence of PLX5622 led to a decrease of IL-4 levels below control levels at 12 h while IL-4 levels were gradually increased above control levels at 96 h. A statistically significant increase in the levels of CXCL1, G-CSF, and MMP-3 was induced in the presence of PLX5622 compared to the control-fed animals challenged with LPS. No differences were observed in the levels of insulin growth factor-1 (IGF-1), monocyte chemoattractant protein 1 (MCP-1), transforming growth factor beta (TGF-beta), interleukin-10 (IL-10), IL-1α, IL-1β, IL-6, macrophage-CSF (M-CSF), tumor necrosis factor-α (TNF-α), macrophage inflammatory protein-1α (MIP-1α), MMP-2, Fas ligand, fractalkine, CXCL1, interferon-γ (IFN- γ), or CCL17.

**Table 2:** Effect of LPS and LPS + PLX5622 in cytokine levels in the blood plasma

	12 h		24 h		96 h	
	LPS	LPS + PLX5622	LPS	LPS + PLX5622	LPS	LPS + PLX5622
CXCL1	1.14	1.40	1.69	4.18***^###^	2.20***	5.02**
G-CSF	12.95***	9.76***^#^	21.94***	30.59***^##^	6.49***	22.72***^##^
IL-4	0.88	0.43**	1.16	1.15	10.31***	16.85***
MMP-3	0.92	0.94	1.82*	3.64***^##^	8.06**	27.62***^###^

All data are normalized to control plasma samples.*p < 0.05, **p < 0.01, ***p < 0.001 compared to control; ^#^*p* < 0.05, ^##^*p* < 0.01, ^###^*p* < 0.001 compared to LPS

### Effect of systemic LPS challenge on microglia proliferation and neutrophils recruitment into the retina

**Fig. 7:**
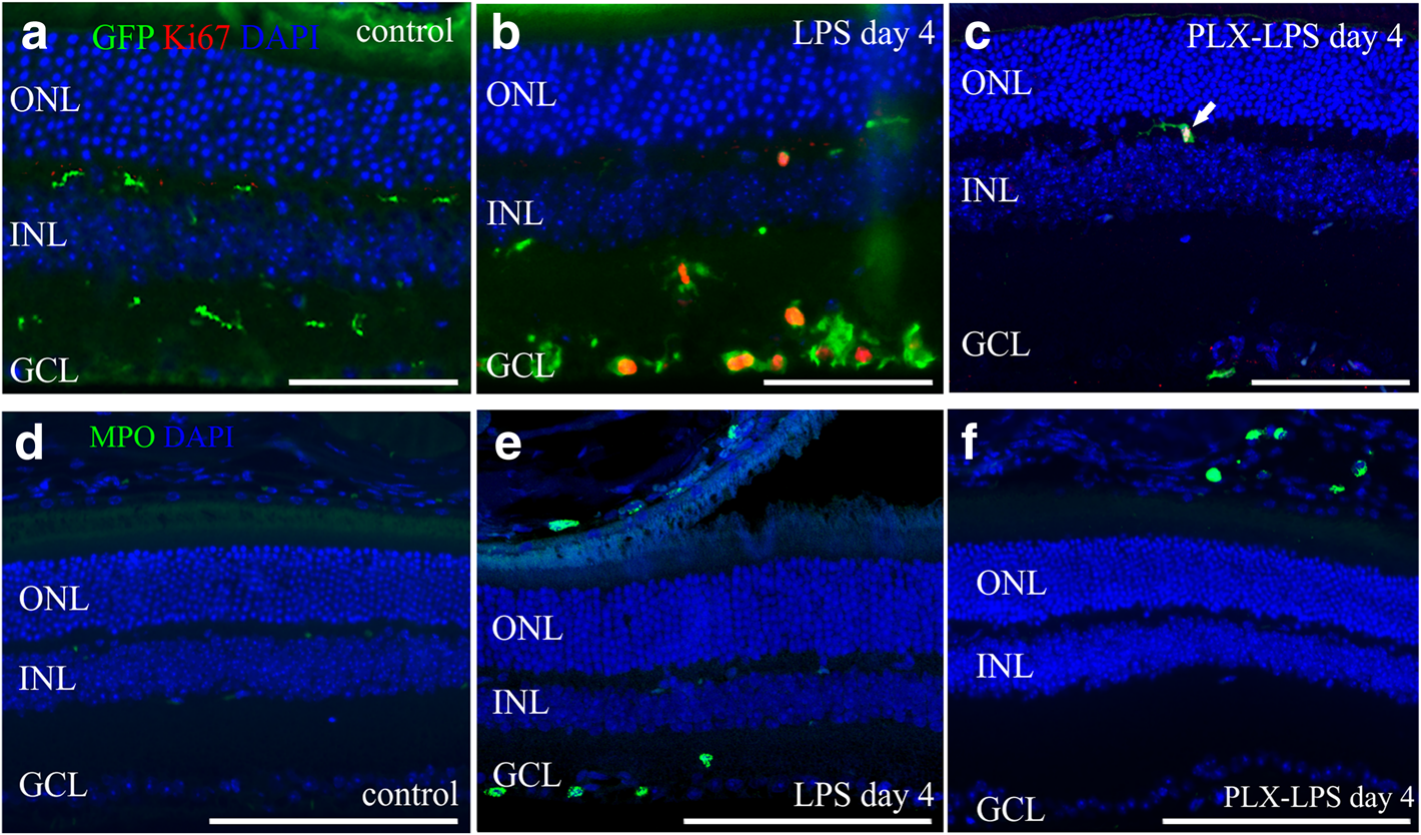
Microglia/macrophages proliferation and neutrophils infiltration upon LPS challenge in the absence or presence of PLX5622. No immunoreactivity of the proliferation marker Ki67 was detected in the control retinas (**a**). Ki67 was co-localized with GFP in retinas of LPS-challenged control-fed (**b**) or PLX-fed mice (**c**). The neutrophil marker myeloperoxidase (MPO) was absent from the retina and choroid of unchallenged mice (**d**). In LPS-challenged mice, MPO was detected into the retina and choroid (**e**), while in the PLX5622-fed LPS-challenged mice MPO was detected in the choroid (**f**) but not into the retina. One representative retina out of five is presented for each group. Scale bars: 100 μm. GCL, ganglion cell layer; INL, inner nuclear layer, ONL, outer nuclear layer

No immunoreactivity of the proliferation marker Ki67 was observed in the control retinas (Fig. 7a), while Ki67 was expressed and co-localized with GFP in the retinas of LPS-challenged mice (Fig. 7b). Isolated proliferating microglia cells were still observed in the retinas of PLX5622-fed mice challenged with LPS (Fig. 7c). The neutrophil marker myeloperoxidase (MPO) was absent in the control eyes (Fig. 7d). MPO positive cells were found in the retina and choroid of the LPS-challenged mice (Fig. 7e) while in PLX5622-fed LPS-challenged mice, MPO-positive neutrophils were exclusively localized in the choroid (Fig. 7f).

## Discussion

Accumulation of sub-retinal fluid is commonly seen in retinal pathologies such as AMD, DR, or uveitis, and the assumed cause is generally agreed to be a breakdown of the outer BRB. The presence of sub-retinal fluid has been correlated with accumulation of activated microglia cells in the sub-retinal space [29, 30]. In the present study, a prolonged systemic inflammatory stimulus with LPS led to increased vascular permeability, retinal swelling, and accumulation of sub-retinal fluid in Balb/c mice (Fig. 1 and Fig. 6q), indicating breakdown of the outer BRB. The BRB is a multicellular vascular structure that separates the neurosensory retina from the peripheral blood circulation and regulates the flux of water and ions into the retina [31], possibly via a family of water membrane-channel proteins, the aquaporins (AQP) [32, 33]. This barrier function prevents leakage of fluid and hydrophilic solutes into the retina and is therefore vital for visual function. The BRB consists of two components; the inner blood-retina barrier constituted by the tight junctions between neighboring retinal endothelial cells, restricting the diffusional permeability of the retinal endothelial layer, and the outer blood-retina barrier, which is formed by the tight junctions between neighboring RPE [33]. The tight junctions of the RPE separate the neural retina from the fenestrated choriocapillaries and are crucial in maintaining retinal adhesion. In our model, the increase in vascular permeability was transient and was accompanied by accumulation of GFP-positive cells around retinal vessels of *Cx3cr1*^*gfp/+*^ LPS-challenged mice (Fig. 2). Thus, we suspect that temporary failure of tight junctions occurred. Moreover, AQP4 expression was not altered in the LPS-challenged mice compared to the control mice (Additional file 1: Figure S1).

In most experimental studies, microglia in the CNS are activated by a single dose of LPS which may bear little resemblance of what occurs in systemic inflammation in humans where bacterial products and toxins can persist for some time. Prolonged LPS exposure may result in permanent microglia activation and increase in vascular permeability. In this line, intestinal-derived endogenous endotoxins such as LPS [34] have been implicated to play an important role in the development of chronic inflammatory conditions such as atherosclerosis which is associated with AMD [35]. In the present study, we show that repeated exposure to LPS leads to activation of microglia/macrophages in the retina (Figs. 2, 3, and 4) peaking at day 4 after the first LPS challenge. Microglia/macrophages numbers were still elevated 3 days after the last LPS injection (Fig. 2), although sub-retinal fluid and fluorescein leakage was not observed at that time point (Fig. 1). Both, microglia as well as macrophage numbers were higher at day 4 after LPS challenge, suggesting on the one hand proliferation of microglia as evident by staining with Ki67 (Fig. 7), as well as migration of circulatory macrophages into the retina revealed by flow cytometry (Fig. 4).

Systemic LPS administration can lead to production of proinflammatory cytokines, such as tumor necrosis factor alpha (TNF-α) or interleukin-1β (IL-1β) from activated microglia in the brain [36, 37] and retina, while it also contributes to the disruption of the blood-brain barrier (BBB) and BRB [38–41]. In line with these findings, we observed a robust increase in a plethora of inflammatory cytokines, including TNF-α, IL-1β, and IFN-γ in the retinas of LPS-challenged mice, 1 day after the challenge (Table 1). However, this increase was transient since after four repetitive LPS injection only basic fibroblast growth factor (bFGF) and VCAM-1 levels were elevated, while the rest of the cytokines examined did not differ from the levels detected in the retinas of unchallenged mice (Table 1).

Repeated peripheral LPS injection can induce so-called endotoxin tolerance [42]. LPS tolerance is thought to result from a hyporesponsive state of microglia/macrophages resulting from receptor desensitization [43, 44] and downregulation of inflammatory cytokine gene expression [45]. However, it has been shown that some inflammatory genes, such as *CCL5*, *CXCL9*, and *CCL8*, remain inducible even after repeated LPS injections [46], while others, such as TNF-α, IL-6, IL-12, IL-1β, CCL3, CCL4, and CXCL10, are downregulated upon LPS re-stimulation. On the other hand, increased secretion of angiogenic factors such as VEGF and bFGF can be detected in tissues after chronic inflammation [47], and this is associated with accumulation of more leukocytes and injury aggravation [48].

The synthesis of proinflammatory cytokines in the central nervous system after peripherally injected LPS has been demonstrated by several studies [49–51] and is further supported by data showing that peripherally released cytokines cannot pass through the intact BBB or BRB [52–54]. Here, BRB breakdown was evident after four but not one LPS challenge. In peripheral blood plasma, only G-CSF was upregulated at the acute phase (Table 2; 12 and 24 h after the first LPS challenge), while after four repetitive LPS injections elevated levels of CXCL1, G-CSF, IL-4, and MMP-3 were detected. Thus, given that the BRB is intact 1 day after the first LPS challenge, we speculate that the robust increase in cytokine levels in the retina represents topical production of cytokines, either from activated microglia/macrophages or from activation of the TRL4 receptors in RPE or retinal endothelial cells [55].

To investigate the role of resident microglia on the LPS-induced phenotype, we employed the highly selective CSF-1R inhibitor PLX5622, which has been previously shown to deplete microglia in the CNS [24, 56]. Accumulation of sub-retinal fluid and retinal swelling were completely abrogated by PLX5622 (Fig. 6), suggesting that the observed breakdown of the BRB after systemic LPS exposure is at least partially mediated by retinal microglia or microglia astrocyte interactions. LPS has been shown to be a strong inducer of astrocytes in the retina [38] and reactive astrocytes have been suggested to play active roles in modulating BRB permeability by release of cytokines such as interleukin-6 (IL-6) and IL-1β [57, 58] and chemokines, such as CX3CL1 and CCL2 [59], while they can be directly activated by LPS via TLR4 receptors [60, 61].

In our study, in the presence of PLX5622, the majority of cytokines induced by LPS returned to control levels, except for a moderate increase on the levels of CCL2, CXCL1, and I-CAM and a decrease in the levels of IL-1α (Table 1). In the blood plasma, the presence of PLX5622 led to elevated levels of CXCL1 compared to LPS, a cytokine that is associated with neutrophil and macrophage maturation and migration [62]. In the present study, we did observe neutrophil accumulation in the choroid of LPS-challenged mice with or without PLX5622 treatment (Fig. 7), while neutrophils were also found within the retinas of LPS-challenged mice but not PLX5622-fed LPS-challenged mice. Due to the preserved integrity of the BRB in the PLX5622-fed mice (Fig. 6), the above suggest that neutrophils are invading into the retina through the disrupted BRB but they do not play a major role in mediating the BRB breakdown induced by LPS.

As mentioned above, PLX5622 has been shown to effectively deplete microglia in the retina and the brain [24, 56] with only modest effects on circulating monocytes and on macrophage numbers in other tissues of wild-type mice [63]. However, in vitro studies have shown that blockade of CSF-1R in bone marrow-derived macrophages renders them resistant to LPS-induced activation [64]. Based on these studies and given the importance of CSF-1R signaling in the differentiation, proliferation, and activation of monocytes [65, 66], one could speculate that PLX5622 may not only affect microglia cell viability in the CNS but also peripheral monocytes’ response to the LPS stimulus, which is subject to further investigation.

## Conclusion

Taken together, the present study provides a useful and highly reproducible model for the study of microglia/macrophage dynamics in retinal diseases influenced by systemic inflammation. Reversal of the LPS-induced phenotype by the CSF-1R inhibitor PLX5622 suggests that microglia and/or infiltrating macrophages participate in the breakdown of the BRB and the accumulation of sub-retinal fluid during chronic inflammation, rendering the regulation of these cells activation a potentially important therapeutic target for retinal disorders where the integrity of the BRB is compromised. However, whether the reduction in cytokine levels and the lack of overt BRB breakdown in PLX5622-fed mice is a direct consequence of the absence of microglial interaction with the BRB, the lack of microglia-mediated astrocyte activation or the failure of monocytes activation remains to be investigated.

## Additional file

Additional file 1: Figure S1 Aquaporin 4 immunoreactivity in the retina of control and LPS-challenged mice. (ZIP 671 kb)

## Abbreviations

AF: Autofluorescence
AMD: Age-related macular degeneration
AQP: Aquaporin
BBB: Blood-brain barrier
bFGF: Basic fibroblast growth factor
BRB: Blood-retina barrier
CCL: CC-chemokine ligand
CNS: Central nervous system
CRG-2: Cytokine-responsive gene-2
CSF-1R: Colony-stimulating factor 1 receptor
CX3CL1: Chemokine (C-X3-C motif) ligand 1
CXCL: Chemokine (C-X-C) motif ligand
EDTA: Ethylenediaminetetraacetic acid
EIU: Endotoxin-induced uveitis
ETDRS: Early Treatment Diabetic Retinopathy Study
FA: Fluorescein angiography
GCL: Ganglion cell layer
G-CSF: Granulocyte colony-stimulating factor
GFP: Green fluorescent protein
GM-CSF: Granulocyte-macrophage colony-stimulating factor
Iba-1: Ionized calcium-binding adapter molecule 1
I-CAM: Intercellular adhesion molecule-1
IFN-γ: Interferon-γ
IGFBP: Insulin-like growth factor-binding protein
IL: Interleukin
INL: Inner nuclear layer
IPL: Inner plexiform layer
LPS: Lipopolysaccharide
M-CSF: Macrophage colony-stimulating factor
MMP-3: Matrix metalloproteinase-3
MPO: Myeloperoxidase
NGS: Normal goat serum
ONL: Outer nuclear layer
OPL: Outer plexiform layer
PBS: Phosphate-buffered saline
PFA: Paraformaldehyde
RPE: Retinal pigment epithelium
SD-OCT: Spectral domain optical coherence tomography
sTNFR: Soluble tumor necrosis factor receptor
TIMP-1α: TIMP metallopeptidase Inhibitor 1α
TLR4: Toll-like receptor 4
TNF-α: Tumor necrosis factor-α
VCAM-1: Vascular cell adhesion molecule-1
VEGF: Vascular endothelial growth factor
XCL1: Chemokine (C motif) ligand

## References

[1] Brito BE, Zamora DO, Bonnah RA, Pan Y, Planck SR, Rosenbaum JT (2004). Toll-like receptor 4 and CD14 expression in human ciliary body and TLR-4 in human iris endothelial cells. Exp Eye Res.

[2] Medeiros R, Rodrigues GB, Figueiredo CP, Rodrigues EB, Grumman A, Menezes-de-Lima O (2008). Molecular mechanisms of topical anti-inflammatory effects of lipoxin A(4) in endotoxin-induced uveitis. Mol Pharmacol.

[3] Chang JH, McCluskey PJ, Wakefield D (2006). Toll-like receptors in ocular immunity and the immunopathogenesis of inflammatory eye disease. Br J Ophthalmol.

[4] Qi Y, Zhao M, Bai Y, Huang L, Yu W, Bian Z (2014). Retinal ischemia/reperfusion injury is mediated by Toll-like receptor 4 activation of NLRP3 inflammasomes. Invest Ophthalmol Vis Sci.

[5] Zech JC, Pouvreau I, Cotinet A, Goureau O, Le Varlet B, de Kozak Y (1998). Effect of cytokines and nitric oxide on tight junctions in cultured rat retinal pigment epithelium. Invest Ophthalmol Vis Sci.

[6] Miyamoto K, Ogura Y, Hamada M, Nishiwaki H, Hiroshiba N, Honda Y (1996). In vivo quantification of leukocyte behavior in the retina during endotoxin-induced uveitis. Invest Ophthalmol Vis Sci.

[7] Nimmerjahn A, Kirchhoff F, Helmchen F (2005). Resting microglial cells are highly dynamic surveillants of brain parenchyma in vivo. Science.

[8] Ebneter A, Kokona D, Schneider N, Zinkernagel MS (2017). Microglia activation and recruitment of circulating macrophages during ischemic experimental branch retinal vein occlusion. Invest Ophthalmol Vis Sci.

[9] Zinkernagel MS, Chinnery HR, Ong ML, Petitjean C, Voigt V, McLenachan S (2013). Interferon gamma-dependent migration of microglial cells in the retina after systemic cytomegalovirus infection. Am J Pathol.

[10] Joly S, Francke M, Ulbricht E, Beck S, Seeliger M, Hirrlinger P (2009). Cooperative phagocytes: resident microglia and bone marrow immigrants remove dead photoreceptors in retinal lesions. Am J Pathol.

[11] El-Remessy AB, Behzadian MA, Abou-Mohamed G, Franklin T, Caldwell RW, Caldwell RB (2003). Experimental diabetes causes breakdown of the blood-retina barrier by a mechanism involving tyrosine nitration and increases in expression of vascular endothelial growth factor and urokinase plasminogen activator receptor. Am J Pathol.

[12] Takeda M, Mori F, Yoshida A, Takamiya A, Nakagomi S, Sato E (2001). Constitutive nitric oxide synthase is associated with retinal vascular permeability in early diabetic rats. Diabetologia.

[13] Leal EC, Manivannan A, Hosoya K, Terasaki T, Cunha-Vaz J, Ambrosio AF (2007). Inducible nitric oxide synthase isoform is a key mediator of leukostasis and blood-retinal barrier breakdown in diabetic retinopathy. Invest Ophthalmol Vis Sci.

[14] Zheng L, Du Y, Miller C, Gubitosi-Klug RA, Kern TS, Ball S (2007). Critical role of inducible nitric oxide synthase in degeneration of retinal capillaries in mice with streptozotocin-induced diabetes. Diabetologia.

[15] Govers R, Oess S (2004). To NO or not to NO: ‘where?’ is the question. Histol Histopathol.

[16] Bal-Price A, Brown GC (2001). Inflammatory neurodegeneration mediated by nitric oxide from activated glia-inhibiting neuronal respiration, causing glutamate release and excitotoxicity. J Neurosci.

[17] Omri S, Behar-Cohen F, de Kozak Y, Sennlaub F, Verissimo LM, Jonet L (2011). Microglia/macrophages migrate through retinal epithelium barrier by a transcellular route in diabetic retinopathy: role of PKCzeta in the Goto Kakizaki rat model. Am J Pathol.

[18] Krady JK, Basu A, Allen CM, Xu Y, LaNoue KF, Gardner TW (2005). Minocycline reduces proinflammatory cytokine expression, microglial activation, and caspase-3 activation in a rodent model of diabetic retinopathy. Diabetes.

[19] Zeiss CJ, Johnson EA (2004). Proliferation of microglia, but not photoreceptors, in the outer nuclear layer of the rd-1 mouse. Invest Ophthalmol Vis Sci.

[20] Appelbaum T, Santana E, Aguirre GD (2017). Strong upregulation of inflammatory genes accompanies photoreceptor demise in canine models of retinal degeneration. PLoS One.

[21] Yoshida N, Ikeda Y, Notomi S, Ishikawa K, Murakami Y, Hisatomi T (2013). Laboratory evidence of sustained chronic inflammatory reaction in retinitis pigmentosa. Ophthalmology.

[22] Zeng HY, Zhu XA, Zhang C, Yang LP, Wu LM, Tso MO (2005). Identification of sequential events and factors associated with microglial activation, migration, and cytotoxicity in retinal degeneration in rd mice. Invest Ophthalmol Vis Sci.

[23] Zeng H, Ding M, Chen XX, Lu Q (2014). Microglial NADPH oxidase activation mediates rod cell death in the retinal degeneration in rd mice. Neuroscience.

[24] Ebneter A, Kokona D, Jovanovic J, Zinkernagel MS (2017). Dramatic effect of oral CSF-1R kinase inhibitor on retinal microglia revealed by in vivo scanning laser ophthalmoscopy. Transl Vis Sci Technol.

[25] Jung S, Aliberti J, Graemmel P, Sunshine MJ, Kreutzberg GW, Sher A (2000). Analysis of fractalkine receptor CX(3)CR1 function by targeted deletion and green fluorescent protein reporter gene insertion. Mol Cell Biol.

[26] Giannakaki-Zimmermann H, Kokona D, Wolf S, Ebneter A, Zinkernagel MS (2016). Optical coherence tomography angiography in mice: comparison with confocal scanning laser microscopy and fluorescein angiography. Transl Vis Sci Technol.

[27] Schneider CA, Rasband WS, Eliceiri KW (2012). NIH image to ImageJ: 25 years of image analysis. Nat Methods.

[28] Kokona D, Schneider N, Giannakaki-Zimmermann H, Jovanovic J, Ebneter A, Zinkernagel M (2017). Noninvasive quantification of retinal microglia using Widefield autofluorescence imaging. Invest Ophthalmol Vis Sci.

[29] Gupta N, Brown KE, Milam AH (2003). Activated microglia in human retinitis pigmentosa, late-onset retinal degeneration, and age-related macular degeneration. Exp Eye Res.

[30] Combadiere C, Feumi C, Raoul W, Keller N, Rodero M, Pezard A (2007). CX3CR1-dependent subretinal microglia cell accumulation is associated with cardinal features of age-related macular degeneration. J Clin Invest.

[31] Strauss O (2005). The retinal pigment epithelium in visual function. Physiol Rev.

[32] Agre P (2004). Aquaporin water channels (Nobel Lecture). Angew Chem.

[33] Shakib M, Cunha-Vaz JG (1966). Studies on the permeability of the blood-retinal barrier. IV Junctional complexes of the retinal vessels and their role in the permeability of the blood-retinal barrier. Exp Eye Res.

[34] Cani PD, Amar J, Iglesias MA, Poggi M, Knauf C, Bastelica D (2007). Metabolic endotoxemia initiates obesity and insulin resistance. Diabetes.

[35] Wong TY, Tikellis G, Sun C, Klein R, Couper DJ, Sharrett AR (2007). Age-related macular degeneration and risk of coronary heart disease: the atherosclerosis risk in communities study. Ophthalmology.

[36] Jeong HK, Jou I, Joe EH (2010). Systemic LPS administration induces brain inflammation but not dopaminergic neuronal death in the substantia nigra. Exp Mol Med.

[37] Qin L, Wu X, Block ML, Liu Y, Breese GR, Hong JS (2007). Systemic LPS causes chronic neuroinflammation and progressive neurodegeneration. Glia.

[38] Ren JL, Yu QX, Liang WC, Leung PY, Ng TK, Chu WK (2018). Green tea extract attenuates LPS-induced retinal inflammation in rats. Sci Rep.

[39] Li X, Gu X, Boyce TM, Zheng M, Reagan AM, Qi H (2014). Caveolin-1 increases proinflammatory chemoattractants and blood-retinal barrier breakdown but decreases leukocyte recruitment in inflammation. Invest Ophthalmol Vis Sci.

[40] Banks WA, Gray AM, Erickson MA, Salameh TS, Damodarasamy M, Sheibani N (2015). Lipopolysaccharide-induced blood-brain barrier disruption: roles of cyclooxygenase, oxidative stress, neuroinflammation, and elements of the neurovascular unit. J Neuroinflammation.

[41] Ghosh A, Birngruber T, Sattler W, Kroath T, Ratzer M, Sinner F (2014). Assessment of blood-brain barrier function and the neuroinflammatory response in the rat brain by using cerebral open flow microperfusion (cOFM). PLoS One.

[42] Wendeln AC, Degenhardt K, Kaurani L, Gertig M, Ulas T, Jain G (2018). Innate immune memory in the brain shapes neurological disease hallmarks. Nature.

[43] Medvedev AE, Kopydlowski KM, Vogel SN (2000). Inhibition of lipopolysaccharide-induced signal transduction in endotoxin-tolerized mouse macrophages: dysregulation of cytokine, chemokine, and toll-like receptor 2 and 4 gene expression. J Immunol.

[44] Dobrovolskaia MA, Medvedev AE, Thomas KE, Cuesta N, Toshchakov V, Ren T (2003). Induction of in vitro reprogramming by Toll-like receptor (TLR)2 and TLR4 agonists in murine macrophages: effects of TLR “homotolerance” versus “heterotolerance” on NF-kappa B signaling pathway components. J Immunol.

[45] Wolk K, Docke WD, von Baehr V, Volk HD, Sabat R (2000). Impaired antigen presentation by human monocytes during endotoxin tolerance. Blood.

[46] Foster SL, Hargreaves DC, Medzhitov R (2007). Gene-specific control of inflammation by TLR-induced chromatin modifications. Nature.

[47] Byrd V, Zhao XM, McKeehan WL, Miller GG, Thomas JW (1996). Expression and functional expansion of fibroblast growth factor receptor T cells in rheumatoid synovium and peripheral blood of patients with rheumatoid arthritis. Arthritis Rheum.

[48] Reinders ME, Sho M, Izawa A, Wang P, Mukhopadhyay D, Koss KE (2003). Proinflammatory functions of vascular endothelial growth factor in alloimmunity. J Clin Invest.

[49] Laye S, Parnet P, Goujon E, Dantzer R (1994). Peripheral administration of lipopolysaccharide induces the expression of cytokine transcripts in the brain and pituitary of mice. Brain Res Mol Brain Res.

[50] Gabellec MM, Griffais R, Fillion G, Haour F (1995). Expression of interleukin 1 alpha, interleukin 1 beta and interleukin 1 receptor antagonist mRNA in mouse brain: regulation by bacterial lipopolysaccharide (LPS) treatment. Brain Res Mol Brain Res.

[51] Pitossi F, del Rey A, Kabiersch A, Besedovsky H (1997). Induction of cytokine transcripts in the central nervous system and pituitary following peripheral administration of endotoxin to mice. J Neurosci Res.

[52] Elmquist JK, Scammell TE, Saper CB (1997). Mechanisms of CNS response to systemic immune challenge: the febrile response. Trends Neurosci.

[53] Vallieres L, Rivest S (1997). Regulation of the genes encoding interleukin-6, its receptor, and gp130 in the rat brain in response to the immune activator lipopolysaccharide and the proinflammatory cytokine interleukin-1beta. J Neurochem.

[54] Lacroix S, Rivest S (1998). Effect of acute systemic inflammatory response and cytokines on the transcription of the genes encoding cyclooxygenase enzymes (COX-1 and COX-2) in the rat brain. J Neurochem.

[55] Tu Z, Portillo JA, Howell S, Bu H, Subauste CS, Al-Ubaidi MR (2011). Photoreceptor cells constitutively express functional TLR4. J Neuroimmunol.

[56] Elmore MR, Najafi AR, Koike MA, Dagher NN, Spangenberg EE, Rice RA (2014). Colony-stimulating factor 1 receptor signaling is necessary for microglia viability, unmasking a microglia progenitor cell in the adult brain. Neuron.

[57] Lee HJ, Suk JE, Patrick C, Bae EJ, Cho JH, Rho S (2010). Direct transfer of alpha-synuclein from neuron to astroglia causes inflammatory responses in synucleinopathies. J Biol Chem.

[58] Nakazawa T, Hisatomi T, Nakazawa C, Noda K, Maruyama K, She H (2007). Monocyte chemoattractant protein 1 mediates retinal detachment-induced photoreceptor apoptosis. Proc Natl Acad Sci U S A.

[59] Farina C, Aloisi F, Meinl E (2007). Astrocytes are active players in cerebral innate immunity. Trends Immunol.

[60] Shen W, Li S, Chung SH, Gillies MC (2010). Retinal vascular changes after glial disruption in rats. J Neurosci Res.

[61] Noailles A, Maneu V, Campello L, Lax P, Cuenca N (2018). Systemic inflammation induced by lipopolysaccharide aggravates inherited retinal dystrophy. Cell Death Dis.

[62] Sawant KV, Poluri KM, Dutta AK, Sepuru KM, Troshkina A, Garofalo RP (2016). Chemokine CXCL1 mediated neutrophil recruitment: role of glycosaminoglycan interactions. Sci Rep.

[63] Mok S, Koya RC, Tsui C, Xu J, Robert L, Wu L, Graeber TG, West BL, Bollag G, Ribas A. Inhibition of CSF-1 receptor improves the antitumor efficacy of adoptive cell transfer immunotherapy. Cancer Res. 2014;74(1):153-61 10.1158/0008-5472.CAN-13-1816 PMC3947337 24247719

[64] Lenda DM, Kikawada E, Stanley ER, Kelley VR (2003). Reduced macrophage recruitment, proliferation, and activation in colony-stimulating factor-1-deficient mice results in decreased tubular apoptosis during renal inflammation. J Immunol.

[65] Guilbert LJ, Stanley ER (1980). Specific interaction of murine colony-stimulating factor with mononuclear phagocytic cells. J Cell Biol.

[66] Byrne PV, Guilbert LJ, Stanley ER (1981). Distribution of cells bearing receptors for a colony-stimulating factor (CSF-1) in murine tissues. J Cell Biol.

