# Supplementary material for: Colony-stimulating factor 1 receptor inhibition prevents disruption of the blood-retina barrier during chronic inflammation
Source: J Neuroinflammation. 2018 Dec 12;15:340. doi: 10.1186/s12974-018-1373-4 (PMC6292111; doi:10.1186/s12974-018-1373-4)
Supplement: Supplementary file 1 — Figure S1 Aquaporin 4 immunoreactivity in the retina of control and LPS-challenged mice. (ZIP 671 kb) [file 12974_2018_1373_MOESM1_ESM.zip › Supplementary information.docx]

**Additional file 1**

**Figure 1: Aquaporin-4 immunoreactivity in the retina of control and LPS-challenged mice.** In control retinas aquaporin-4 (AQP-4) immunoreactivity was localized throughout the retina and predominantly in the inner retinal layers (**a**). No difference was observed in the expression pattern of AQP4 in retinas of LPS-challenged mice (**b**). One representative image out of 5 is presented for each group. Scale bars: 100 μm. GCL, ganglion cell layer; INL, inner plexiform layer; IPL, inner plexiform layer; ONL, outer nuclear layer.
