# Supplementary figures and images for: Colony-stimulating factor 1 receptor inhibition prevents disruption of the blood-retina barrier during chronic inflammation
Source: J Neuroinflammation. 2018 Dec 12;15:340. doi: 10.1186/s12974-018-1373-4 (PMC6292111; doi:10.1186/s12974-018-1373-4)

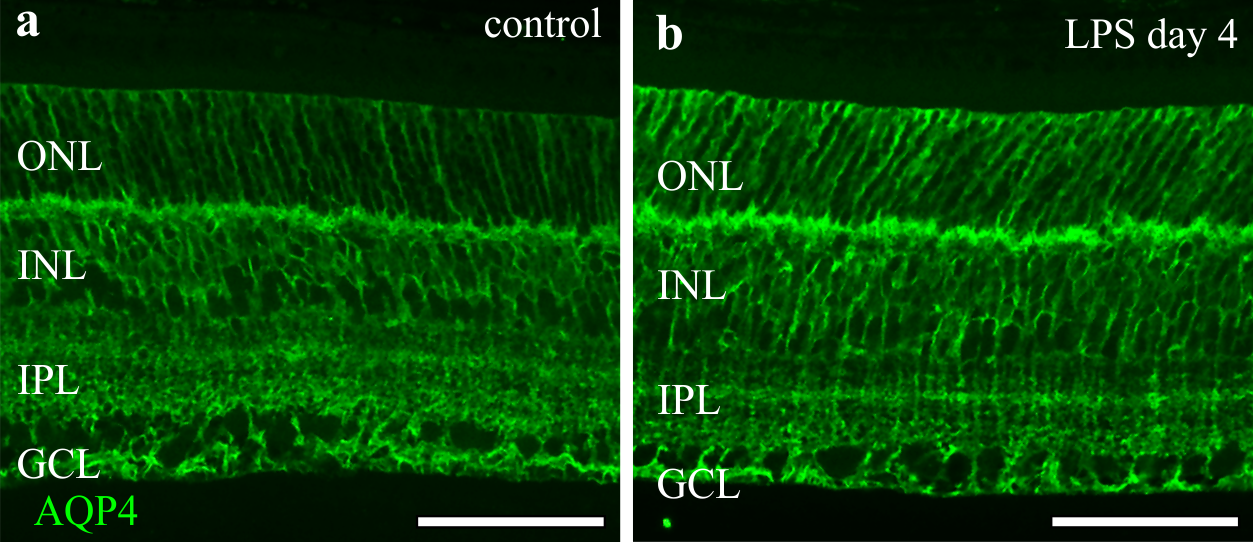

Supplement: Supplementary file 1 — Figure S1 Aquaporin 4 immunoreactivity in the retina of control and LPS-challenged mice. (ZIP 671 kb) [file 12974_2018_1373_MOESM1_ESM.zip › Supplementary Figure 1.tif]
